# Spectral EEG in Congenital Heart Disease: A Case–Control Study in Infants Undergoing Cardiac Surgery

**DOI:** 10.1007/s00246-025-03958-7

**Published:** 2025-07-25

**Authors:** Elisa Cainelli, Manuela Simonato, Anna Sartori, Massimo Padalino, Stefano Sartori, Claudio Ancona, Luca Vedovelli, Paola Cogo, Patrizia Bisiacchi

**Affiliations:** 1 Department of General Psychology, University of Padova, Via Venezia, 8, 35133 Padua, Italy; 2 PCare Laboratory, Fondazione Istituto Di Ricerca Pediatrica “Citta Della Speranza”, Padua, Italy; 3 Pediatric and Congenital Cardiovascular Surgery Unit, Department of Cardiac, Thoracic and Vascular Sciences, Padova University Hospital, Padua, Italy; 4 Paediatric Neurology and Neurophysiology Unit, Department of Women’s and Children’s Health, University Hospital of Padova, Padua, Italy; 5 Neuroimmunology Group, Paediatric Research Institute “Città Della Speranza”, Padua, Italy; 6 Department of Neuroscience, University of Padova, Padua, Italy; 7 Unit of Biostatistics, Epidemiology, and Public Health, Department of Cardiac, Thoracic, Vascular Sciences, and Public Health, University of Padova, Padua, Italy; 8 Department of Medicine, Clinica Pediatrica, University Hospital S Maria Della Misericordia, University of Udine, Udine, Italy

**Keywords:** Power spectrum, Psychomotor, Griffith, Frequency, Neurodevelopment

## Abstract

Children suffering from congenital heart disease (CHD) are at risk of developing a variety of neurocognitive sequelae. The etiology of these impairments is thought to be multifactorial, but the underlying mechanisms are poorly understood. The aim of this study was to compare the power spectra of postoperative electroencephalographic (EEG) activity of children with CHD undergoing cardiac surgery and controls to highlight differences in brain oscillations and cerebral architecture. We recruited 35 children with CHD undergoing cardiac surgery within six months of life (CHD group, *M* = 22, age at evaluation 111 days [38; 173]) and 35 age-matched healthy controls (control group, *M* = 24). After surgery, immediately before discharge, CHD children underwent multichannel EEG and psychomotor evaluation with the Griffiths Mental Developmental Scale (GMDS). At the psychomotor evaluation, 25/35 (71%) children in CHD group had a normal GMDS total score, 7/35 (20%) were borderline, and 3/35 (5%) were impaired. The EEG activity of CHD and controls differed significantly in the alpha (*F* = 23.662, *p* < .001) and beta (*F* = 36.457, *p* < .001) bands. Only for the CHD group did EEG power not correlate with age. CHD children exhibit marked differences in the EEG spectrum, particularly in the medium–high frequencies, suggesting an abnormal development of cerebral networks sustaining early cognitive milestones.

## Background

Congenital heart diseases (CHDs) are the most common congenital defects, affecting nearly 1% of all newborns [1]. Although advances in medical care have significantly improved survival rates, long-term cognitive deficits have been reported, particularly in patients undergoing cardiac surgery, due to the increased risk of brain injury [2–5]. Even in the absence of major neuromotor impairments or intellectual disability, children may exhibit subtle, isolated neuropsychological difficulties and psychological disorders [2, 6, 7], with high variability in outcomes [8], and a significant impact on social life, academic performance, and socioeconomic well-being in adulthood [9].

These deficits are believed to have a multifactorial pathogenesis, involving both prenatal and postnatal factors. In addition to the specific cardiac morphology, various acquired factors may contribute, such as fluctuations in cerebral blood flow and oxygenation, the degree of hypothermia applied during cardiopulmonary bypass (CPB) [10], and variations in intravascular volume that can alter cerebral hemodynamics in the early postoperative period [11].

Unfortunately, the exact neurofunctional mechanisms underlying the development of neurocognitive disabilities remain poorly understood [12, 13], particularly in patients without overt brain injury on neuroimaging [14, 15]. In the absence of structural brain damage, neurofunctional methodologies become especially important. Compared to neuroimaging, neurophysiological techniques such as electroencephalography (EEG) provide unique insights into brain function, detecting functional abnormalities that may not be visible with structural imaging methods.

Most studies investigating neurofunctional methods in children with CHD have focused on qualitative visual EEG analysis. While visual EEG interpretation is a valuable predictive tool in severe cases (e.g., in the presence of seizures or a markedly depressed background), it lacks sensitivity in milder cases, where subtle abnormalities in cerebral oscillations may reflect an underlying neural vulnerability [16, 17]. Additionally, qualitative EEG analysis requires expert interpretation by experienced neurophysiologists.

Quantitative EEG methods, including coherence and connectivity analysis, have highlighted early disruptions in developing neuronal networks that, if persistent, may contribute to long-term neurocognitive impairments in children with CHD [18, 19]. However, there is limited information available on EEG spectral power—the quantification of brain oscillations across different frequency bands—which can provide important insights into cerebral circuit development, detect abnormal patterns, and help predict future outcomes [19].

The aim of the present study is to compare EEG spectral power in children with CHD who underwent cardiac surgery (CHD group) with that of healthy controls (control group). We also evaluated the influence of clinical characteristics and surgical factors on EEG patterns and psychomotor development within the CHD group.

## Methods

### Participants

All infants undergoing cardiac surgery for CHD between March 2021 and June 2024 at the Pediatric Cardiac Anesthesia/Intensive Care Unit and Pediatric Cardiothoracic Surgery Unit, Centro Gallucci of the University of Padova, were recruited prospectively (CHD group) if they met the following inclusion criteria: infants with complex biventricular CHD requiring elective cardiac surgery within 6 months of life, with CPB time > 90 min, aortic cross-clamping (ACC) > 30 min, and written informed consent. Exclusion criteria: previous heart surgery, other associated malformations or genetic anomalies, prematurity (≤ 37 weeks of gestational age), hemodynamic instability requiring inotropic support and/or mechanical ventilation, factor V < 20%, or creatinine clearance < 30% before surgery, as signs of renal and hepatic failure, respectively. In addition, before surgery, all infants underwent a neurological evaluation, head ultrasound and MRI if clinically indicated. Infants with documented strokes or brain hemorrhage before cardiac surgery by head ultrasound and MRI or being treated with drugs (e.g., sedatives) affecting the central nervous system were excluded.

Demographic and preoperative data were recorded, including mode of delivery, birth weight, gestational age, Apgar score, balloon atrial septostomy, low cardiac output syndrome, and preoperative intensive care time; CHD was classified according to the Society of Thoracic Surgeons-European Association for Cardio-Thoracic Surgery (STAT) score [20].

The following intra and postoperative parameters have been recorded: age in days at surgery, duration of CPB perfusion, ACC, hypothermia and surgery, circulatory arrest or selective cerebral perfusion, dose and timing of anesthetic drugs, and blood gases. After surgery, we recorded sedation and analgesic drugs, postoperative infections (culture proven), arrhythmia and low cardiac output state, and cerebral complications (seizures, bleeding, strokes) that occurred either during the hospital stay or at the clinical follow-up.

Control group included age-matched controls recruited from a database of subjects who underwent EEG at the Pediatric Neurology and Neurophysiology Unit of the Department of Women’s and Children’s Health of the University of Padua from 2013 to 2020 after a paroxysmal event. Once the non-epileptic nature of the event had been ascertained, subjects were recruited based on the following inclusion criteria: normal neurodevelopment and no ongoing neurological issues*;* normal EEG tracings; and the presence of at least 5 min of quiet wakefulness free of artifacts. Exclusion criteria were as follows*:* a pathological EEG in the previous six months; diagnosis of a neurological condition, neurodevelopmental disorder, or psychiatric disease; and ongoing therapy with benzodiazepines, phenobarbital, steroids, or other drugs potentially interfering with the EEG signal.

### EEG

Recordings were performed after surgery immediately before discharge from the hospital when infants were clinically stable. A 9-channel cap (Fp1, Fp2, C3, C4, Cz, T3, T4, O1, and O2) adapted for neonates and infants has been used. The ground electrode was placed at Fpz and the reference to earlobes. Impedance was less than 10 *K*Ω and balanced in all electrodes. We used a Micromed SD MRI 64 system (Micromed s.p.a., Treviso, Italy). Digital EEG signals were sampled at 512 Hz and stored on a hard disk. Artifacts related to the infant’s movements or replacement of an electrode were removed when the impedance value was > 10 *k*Ω. We analyzed EEG segments recorded in quiet wakefulness. For offline analysis, five minutes of artifact-free EEG traces recorded in quiet wakefulness were selected.

Pre-processing and spectral analysis were performed using the EEGLAB toolbox and a custom-scripted software in the MATLAB environment. Data were filtered using 0.5 Hz high-pass and 20 Hz low-pass filters. After multiplication with a Hanning window function, the time series were transformed into the frequency domain using an efficient Fast Fourier Transform algorithm. Frequency spectrum and frequency band powers were determined for each 2-s segment (50% overlapping) and averaged over the complete five-minute recording to obtain mean EEG frequency parameters per EEG recording. The frequency spectrum was divided into the following bands: delta (0.5–4 Hz), theta (4–7 Hz), alpha (8–13 Hz), and beta (14–20 Hz). The absolute power (defined as the integral of all powers within the frequency band, expressed in *µ*V2) was calculated from the transformed signal. As the total absolute spectral power may vary considerably, spectral values among subjects were normalized for total power and expressed as relative spectral power measures (defined as the ratio of absolute band power and total power fall band, expressed in percentage). The mean values across the channel have been calculated to obtain a global measure across the 4 frequency bands.

All children had discontinued sedative/anesthetic medication used during the surgery and the intensive therapy unit (morphine, fentanyl, midazolam, propofol, ketamine, dexmedetomidine, cisatracurium, atracurium, and Sevoflurane) at least 5 days prior the time of the EEG and GMDS recordings.

### Griffiths Mental Developmental Scales (GMDS)

On the same day as the EEG recording, a chartered psychologist evaluated the psychomotor development using the Griffiths Mental Developmental Scales (GMDS, [21]). The scales were scientifically constructed based on item analysis and developmental theory and chosen for their clinical utility, psychometric properties, and validity. Furthermore, the GMDS demonstrated continuing validity over time and across cultures [22].

The GMDS provides a simple ratio transformation, dividing the mental age by chronological age, yielding means and standard deviations for the global quotient and six subscale scores: Locomotor, Personal-social, Language, Eye-hand Coordination, Performance tests, and Practical Reasoning (after 2 years) [23]. The locomotor subscale (a) measures in detail all the developing skills involved in achieving the upright posture and lead to walking, running, and climbing. The personal–social subscale (b) measures the development of personal autonomies, emotional regulation, and social skills. The language subscale (c) evaluates hearing, in the sense of active listening, and the child’s first progress in acquiring a vocabulary of sounds, vocalization, and bubble or prespeech, which is finally superseded by adult language. The hand–eye coordination subscale (d) assesses the child’s level of manipulation, fine motor skills, manual dexterity, and visual monitoring skills. Finally, the performance subscale (e) includes a series of performance tests, that assess the developing ability to reason in practical situations or manipulate materials’ intelligently (e.g., placing insets in the correct hole of a form board, completing a set of boxes containing bricks, finding hidden objects). After 2 years, the scale adds the subscale (f) of practical reasoning.

The obtained quotients were considered normal between 85 and 115, borderline if they fell between 85 and 70, and abnormal below 70.

### Statistical Analysis

EEG spectral power in the delta, theta, alpha, and beta of control and CHD groups, with age as a covariate, has been compared using a multivariate ANOVA. The TGA patients were the only subgroup with a median age in the neonatal period; therefore, they warranted a separate analysis given expected differences in EEG spectra in this age group compared to infants. Thus, to exclude the potential confounding effect of the different ages of the TGA group, the analysis has been run separately for TGA versus matched controls, and CHD non-TGA versus matched controls. Finally, the groups TGA versus non-TGA have been compared.

The effect of clinical parameters (surgery length, hypothermia length, rewarming time during CPB, CEC length, time elapsed by sedative and analgesic medication interruption) on EEG frequency bands and GMDS has been evaluated using distinct linear regression models. The correlations between EEG and GMDS were determined using a non-parametric Spearman rho.

Statistics were determined using JASP (The JASP team 2018, version 0.19.2).

## Results

### Descriptive Data

Our final sample consisted of 35 children with CHD (CHD group, males = 22) and 35 age-matched controls (control group, males = 24). In CHD group, 8/35 had a transposition of the great artery (TGA) (TGA subgroup, median age at evaluation = 21 days, Q1 = 20; Q3 = 70.5), 5/35 intraventricular defect (DIV) (DIV subgroup, median age at evaluation = 173 days, Q1 = 164; Q3 = 229.5), 8/35 tetralogy of Fallot (TOF) (TOF subgroup, median age at evaluation = 163 days, Q1 = 130.75; Q3 = 200), 14/35 other cardiac conditions (“Other” subgroup median age at evaluation = 71 days, Q1 = 52.25; Q3 = 141.50). The age didn’t differ significantly between CHD subgroups (Chi-square).

### GMDS Results (CHD Group)

**Fig. 1:**
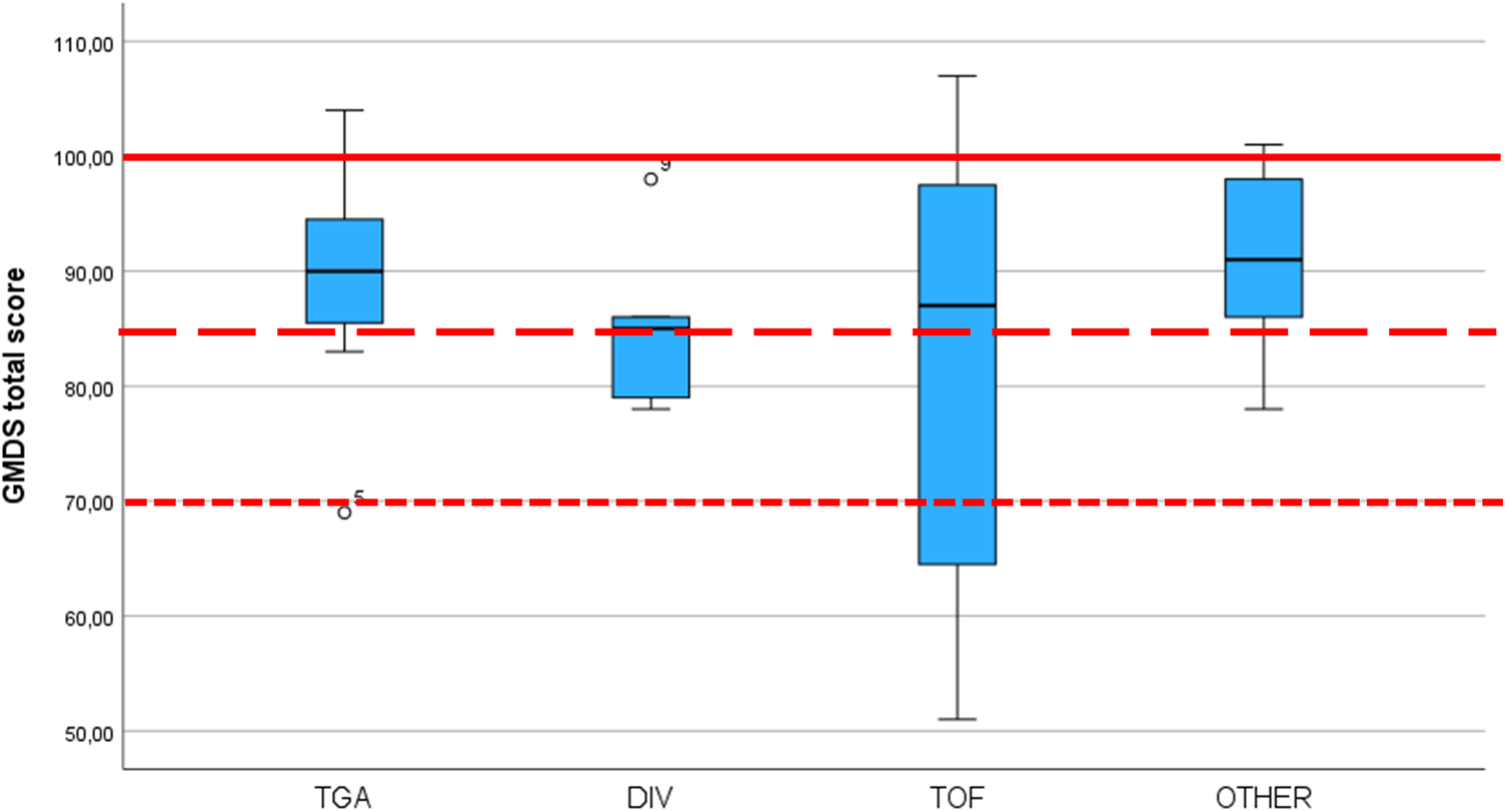
Boxplot of the total GMDS scores in the different CHD subgroups

25/35 (71%) CHD children had a normal score at the GMDS total score, 7/35 (20%) borderline, and 3/35 (5%) impaired. The median scores obtained at the GMDS evaluation show that scores globally fail in the low-normal/borderline range (Fig. 1). The median and quartiles of the CHD group and for CHD subgroups are shown in Table 1.

**Table 1:** Median and quartile scores at the GMDS in the CHD children and for CHD subgroups

GMDS	CHD *N* = 35	TGA *N* = 8	DIV *N* = 5	TOF *N* = 8	Other *N* = 14
Locomotor	87(76; 98)	89.5 (84; 99.7)	76 (69; 84)	87.5(60.5; 96.5)	91 (83.7; 99)
Pers./Social	93(87; 103)	92 (81.5; 102)	87 (81.5; 97.5)	89.5 (68.7; 103)	94(92.7;104)
Language	94 (87; 100)	93 (88.2; 99.7)	93 (88.5; 98.5)	90.5(66;100.7)	94(90.2;100)
Eye/Hand C	87 (79; 98)	86 (80; 96.75)	85 (76; 95)	85 (65.5; 96.2)	88 (82.5; 98)
Performance	86 (79; 95)	84.5(74;95.2)	86 (81; 91.5)	83 (63.2; 99)	89 (79.7; 97)
Total	89 (83; 98)	90 (84.2; 96.2)	85 (78.5; 92)	87 (59.2; 98.25)	91(85;98.2)

The linear regression indicated the duration of the surgery (beta = − 0.144, p = 0.007) and rewarming (beta = 0.305, *p* = 0.012) as independent predictors of the GMDS score.

## EEG results

**Fig. 2:**
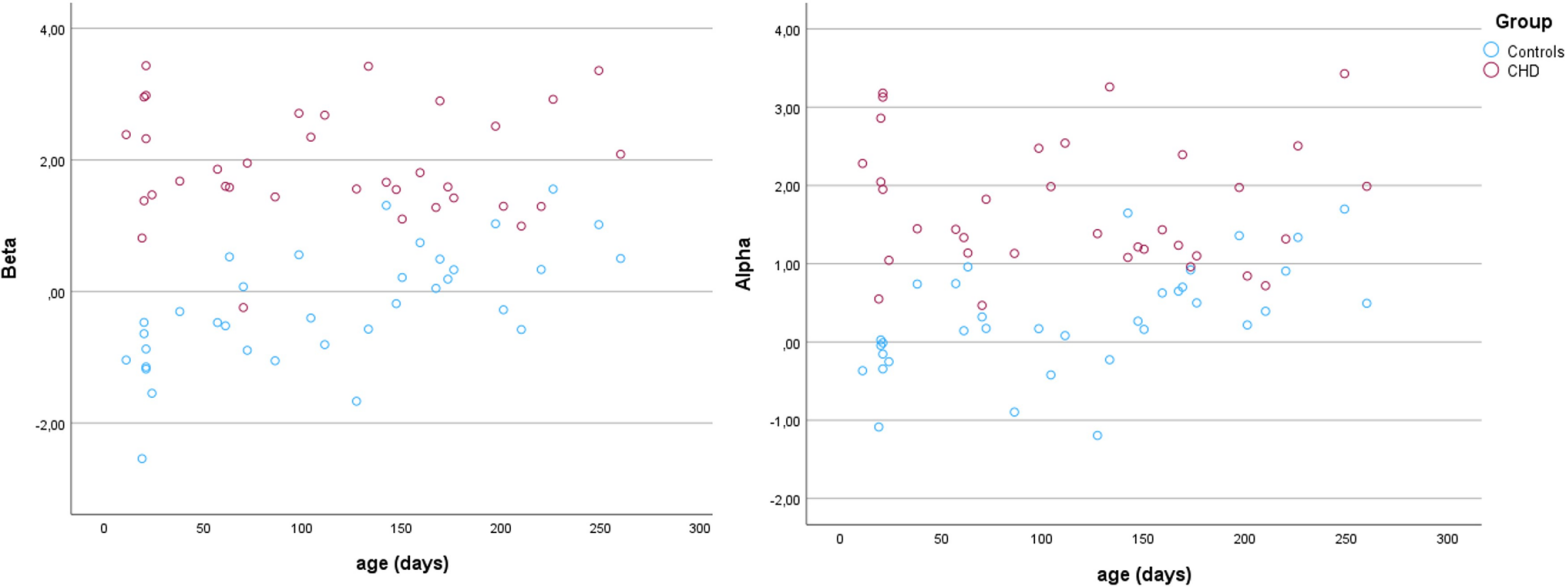
Scatterplot of the power spectra values in the alpha (panel A) and beta (panel B) of the CHD (red) and controls (blue) groups

The multivariate analysis shows that CHD and control groups differed significantly in the alpha (*F* = 23.662, *p* < 0.001) and beta (*F* = 36.457, *p* < 0.001) bands. The scatterplot of the spectral power obtained in the alpha and beta bands for CHD (red) and controls (blue) is shown in Fig. 2.

**Fig. 3:**
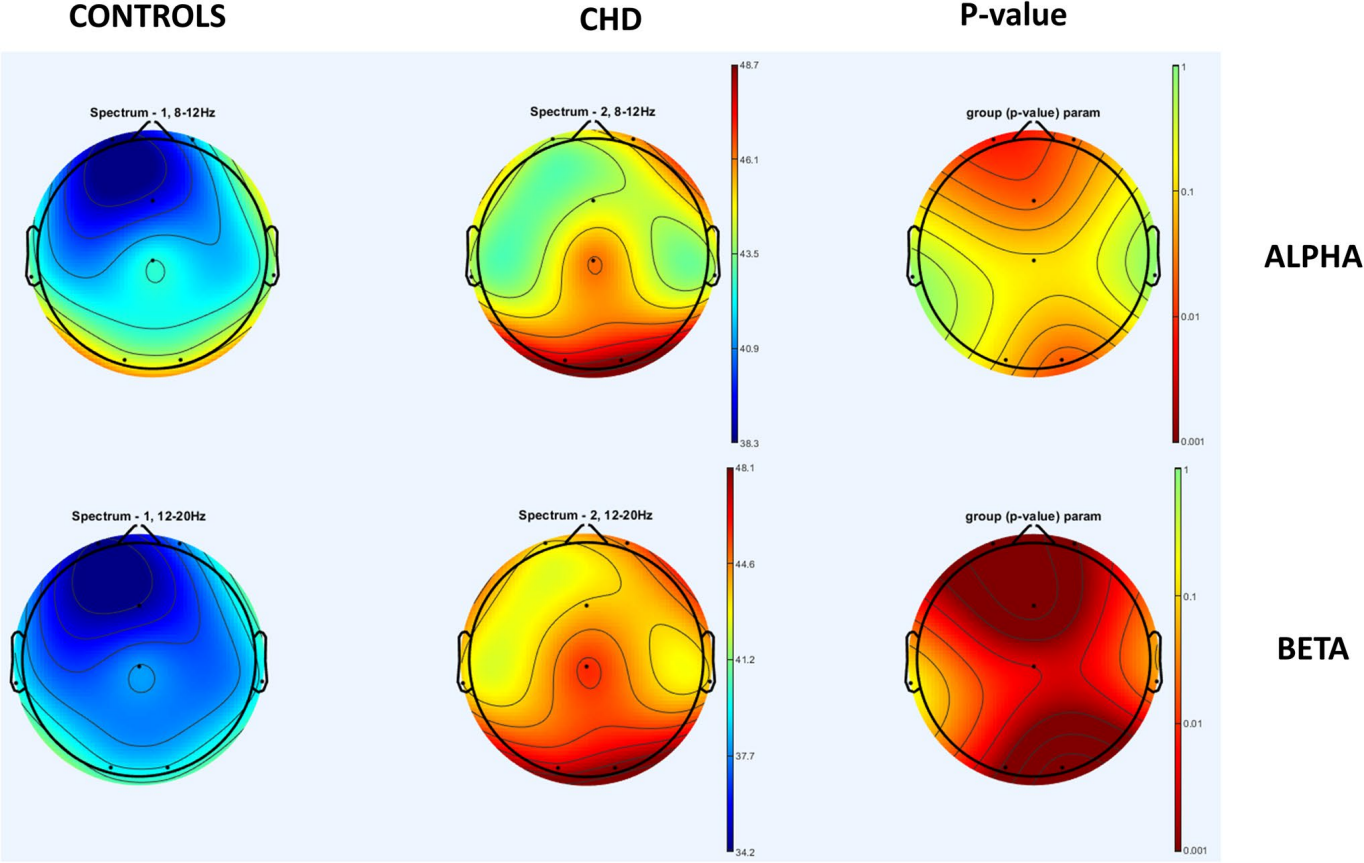
The power spectra in the alpha and beta frequency range on all electrodes in CHD and controls

Figure 3 shows the power in the alpha and beta frequency range on all electrodes in CHD and controls and the relative difference in power, particularly pronounced in the occipital and frontal areas.

Age was significantly associated only with the theta band (*F* = 4.095, *p* = 0.047). When considering the age separately for the two groups, an association was found only in the control group for the theta, alpha, and beta bands (Table 2).

**Table 2:** Spearman correlation between age and EEG spectra power in the CHD and control groups

AGE	CHD		Controls	
	rho	p	Rho	*P*
Delta power	ns		Ns	
Theta power	ns		.600 (.310;.796)	<.001
Alpha power	ns		.613 (337;.797)	<.001
Beta power	ns		.672 (.451;.809)	<.001

The differences between groups remain significant also by comparing the CHD subtypes in isolation; in particular, in the comparison of TGA versus controls, we found significant differences in the alpha (*F* = 7.16, *p* = 0.019) and beta (*F* = 7.18, *p* = 0.018) bands. In the comparison of non-TGA versus controls, we found significant differences in the alpha (*F* = 16.339, *p* < 0.001) and beta (*F* = 29.156, *p* < 0.001) bands.

Furthermore, the groups TGA versus non-TGA did not differ in the EEG spectra.

Neither clinical parameter resulted in a predictor of EEG spectra bands, nor was age at evaluation and the time elapsed from surgery. Finally, we didn’t find significant associations between GMDS and EEG scores.

## Discussion

In this study, we compared the EEG spectral power of infants with congenital heart disease (CHD) after cardiac surgery to that of healthy controls. We found significant differences between the two groups in the percentages of alpha and beta frequencies. Furthermore, the EEG spectra of the CHD group did not show the typical age-related changes that characterize normal neurodevelopment. Psychomotor development assessments revealed that 7 out of 35 CHD children (20%) had borderline GMDS total scores, while 3 out of 35 (5%) had impaired results. However, GMDS scores did not correlate with the EEG.

The increased power in the medium-to-high frequency ranges in the CHD group was sustained and particularly pronounced in some children compared to controls. The origin of this abnormal increase is unclear. A similar pattern of diffuse frequencies, ranging from high alpha to low beta, is typically seen with certain sedative medications, particularly benzodiazepines and barbiturates [24]. However, in the CHD group, all children had discontinued sedatives at least five days prior to EEG recording, making a drug effect or rebound unlikely.

This increase in medium–high frequencies is probably generated by abnormal cerebral oscillations. This may also explain the absence of typical maturational changes in the ratio between frequency bands, which are usually evident during the first year of life. In normal development, the predominant low frequencies characteristic of the neonatal period are gradually replaced by emerging higher frequencies [25, 26]. This trend was evident in the control group, where increases in theta, alpha, and beta bands were significantly associated with age.

Several previous studies have investigated the role of EEG in detecting brain injury or delayed maturation (for a review, see [27]). However, these studies often rely on qualitative visual EEG analysis or amplitude-integrated EEG, which provide different information compared to our spectral power-based methodology. Studies using spectral power to estimate coherence or connectivity have found increased high-frequency connectivity. Markus et al. (2021) observed a distinct emergence of alpha and beta activity, interpreted as an increased inhibitory neuronal function [28]. In another study, a similar increase in high-frequency EEG connectivity was associated with brain injury and immaturity [18]. High-frequency oscillations are thought to support local processing, segregation, and differentiation within specialized cortical regions, while low frequencies support global, distributed processing and integration between distant cortical areas [29].

Therefore, increased high-frequency connectivity in CHD neonates may suggest stronger cortical differentiation but reduced integration across distant brain areas [18]. We didn’t measure connectivity, but in our patients, the high proportion of high alpha/low beta activity could represent a neurophysiological signature of an imbalance in the “local vs. distributed” organization of spontaneous brain networks, possibly reflecting compensatory mechanisms. This shift in frequency content may also indicate a reduced response of local neural populations to global modulatory influences, which are crucial for large-scale integration [25].

The origin of this high-frequency increase remains unclear, in part because a major limitation of our study is the lack of pre-surgery EEG recordings. Previous literature comparing pre- and post-surgical EEGs, mainly based on visual inspection, found a variable percentage of abnormalities before surgery and a marked increase afterward [30–32], suggesting a significant role of surgery and related procedures in generating EEG abnormalities.

The incidence of EEG abnormalities varies across studies [30, 31, 33–35], likely reflecting the complex and heterogeneous etiologies underlying brain dysfunction. CHD infants are at risk of hypoxia, neuroinflammation, stress, and procedures involving general anesthesia. There is evidence that CHD and its associated factors can lead to early structural and microstructural brain abnormalities, with immediate functional consequences observable as altered neuronal network connectivity (Birca). The elusive yet crucial ability of EEG to detect such abnormalities is supported by its predictive power for later cognitive outcomes (see [36] for a review).

Efficient fine-tuning between local and distributed networks is crucial for cognitive development and it is reflected in the balance of high- and low-frequency EEG components. Alterations in this balance may predispose the CHD brain to later neurocognitive and neuropsychological impairments. Early disruption of neuronal network development, if sustained, could underlie the persistent cognitive deficits observed in CHD survivors [37]. Even in the absence of overt brain damage, subtle functional abnormalities in developing circuits may interfere with cognitive milestone acquisition. Moreover, this could impact the maturation of later-developing networks, particularly fronto-subcortical circuits critical for higher cognitive and psychological functions [38].

Thus, in the absence of targeted interventions, disrupted brain circuits may accumulate during development, leading to a cascade of adverse effects on later cognitive functioning. Once consolidated, an altered pattern of functioning may become a stable trait, as shown by long-term follow-up studies in adolescence and adulthood [39, 40]. In children with CHD, cognitive difficulties have been reported throughout infancy, childhood, and adolescence [5]. Early language acquisition and behavioral self-regulation issues [30, 41, 42] may evolve into academic, cognitive, and socio-emotional challenges in school-age children. Specific difficulties in attention, executive functioning, visuospatial processing, processing speed, and social cognition have been documented [2, 4, 6, 43]. Children with CHD are also at higher risk for psychiatric and neurodevelopmental disorders, including mood and anxiety disorders, ADHD, autism spectrum disorder, and learning disabilities [44–47]. Furthermore, even patients with average intelligence and no motor impairments may present subtle neuropsychological difficulties or psychological disorders [48, 49]. These impairments can negatively impact academic performance and social life, leading to long-term effects on adult socioeconomic status and career outcomes [9].

However, brain maturation, including network refinement and myelination, continues throughout childhood, providing a significant opportunity for recovery. This underscores the importance of a lifespan approach to improving outcomes in CHD patients and the importance of supporting the achievement of early developmental milestones that underlie the maturation of more complex cognitive abilities.

Evaluating the emergence and characteristics of spectral EEG components—and their deviations from the expected developmental trajectory—may be crucial for understanding early brain development abnormalities. In a previous study on children with a history of prematurity, we found an association between neonatal spectral EEG data and neurocognitive performance at six years of age, suggesting that spectral features may serve as a useful risk biomarker [50]. As a confirmation of the usefulness of neurophysiological techniques, we found a predictive ability also in other clinical settings and with other methodologies similar to the EEG [51–53]. In the present study, results seem to support the potential role of spectral EEG, although only long-term follow-up will clarify the clinical significance of the identified differences. Once at-risk children are identified, they could be placed in a more stringent and longer neurological and neuropsychological monitoring program. Viewed from a clinical practice perspective, performing an EEG is inexpensive and non-invasive. Using spectra analysis would also mean that specialist personnel would not be required, as it would be easily quantified. Detailed clinical evaluations with a focus on neuropsychological and psychological domains offer promising avenues for future neurological and neurobiological research, even in the challenging population of CHD infants without overt brain injury, in which prognosis is particularly difficult.

In the present study, most children (71%) demonstrated normal psychomotor development. However, the young age of the participants may have limited the sensitivity of the GMDS in detecting subtle atypical developmental trajectories, as noted in other clinical contexts [51]. This may also explain the lack of association between EEG findings and GMDS scores. Nonetheless, GMDS scores were associated with certain clinical factors; in particular, poor GMDS outcome was associated with longer surgery durations and shorter rewarming times during cardiopulmonary bypass (CPB). Longer surgery time may reflect more severe medical conditions or may itself contribute to developmental delays due to the cumulative impact of procedures and associated stressors on the brain.

This study several other limitations. The control group was recruited retrospectively rather than prospectively, requiring the use of normative samples for GMDS interpretation. Despite experiencing hospitalization for non-CHD conditions, the control group may still differ in key aspects such as hospitalization stress, unfamiliar environments, or anxiety. The same EEG methodology was, however, used for both groups. Additionally, our CHD cohort was clinically heterogeneous and too small to examine the effects of multiple clinical variables. Important insights into CHD-related brain development could be provided in future research by comparing CHD patients with other clinical populations (e.g., preterm infants). Thus, regarding the evaluations, only a single EEG and GMDS assessment was conducted, and the study lacked longitudinal follow-up.

Finally, MRI was not performed in all patients, raising the possibility that certain abnormalities—such as subtle signs of cerebral immaturity or microhemorrhages, which may be less evident on cranial ultrasound but potentially relevant to EEG spectral changes—were not documented. Future studies would benefit from investigating the relationship between EEG abnormalities and MRI findings.

## Conclusions

In conclusion, although our group of CHD infants did not show marked neurodevelopmental delays in the first six months of life, they exhibited a significant increase in medium-to-high EEG frequencies, suggesting early abnormal cerebral network organization. The early formation of long- and short-range intracortical connections provides the structural foundation for later cognitive function. Understanding these early disruptions is critical, as key cognitive milestones emerge in early life, while full brain maturation spans decades. Abnormalities in early organization may remain latent for years, making early detection and monitoring essential.

## Data Availability

Data will made available on request.

## Abbreviations

ACC: Aortic cross-clamping
CPB: Cardiopulmonary bypass
CHD: Congenital heart disease
DIV: Intraventricular defect
EEG: Electroencephalogram
GMDS: Griffiths mental developmental scales
STAT: Society of thoracic surgeons-european association for cardio-thoracic surgery
TGA: Transposition of the great artery
TOF: Tetralogy of Fallot
